# How Fear Memory is Updated: From Reconsolidation to Extinction?

**DOI:** 10.1007/s12264-025-01367-7

**Published:** 2025-04-09

**Authors:** Jiahui Chen, Zhuowen Fang, Xiaolan Zhang, Yanrong Zheng, Zhong Chen

**Affiliations:** 1 Zhejiang Key Laboratory of Neuropsychopharmacology, Institute of Pharmacology & Toxicology, College of Pharmaceutical Sciences, Zhejiang University, Hangzhou, 310058 China; 2 Key Laboratory of Neuropharmacology and Translational Medicine of Zhejiang Province, School of Pharmaceutical Sciences, Zhejiang Chinese Medical University, Hangzhou, 310053 China

**Keywords:** Post-traumatic stress disorder, Fear memory, Reconsolidation, Extinction, Engram

## Abstract

Post-traumatic stress disorder (PTSD) is a psychiatric disorder caused by traumatic past experiences, rooted in the neurocircuits of fear memory formation. Memory processes include encoding, storing, and recalling to forgetting, suggesting the potential to erase fear memories through timely interventions. Conventional strategies such as medications or electroconvulsive therapy often fail to provide permanent relief and come with significant side-effects. This review explores how fear memory may be erased, particularly focusing on the mnemonic phases of reconsolidation and extinction. Reconsolidation strengthens memory, while extinction weakens it. Interfering with memory reconsolidation could diminish the fear response. Alternatively, the extinction of acquired memory could reduce the fear memory response. This review summarizes experimental animal models of PTSD, examines the nature and epidemiology of reconsolidation to extinction, and discusses current behavioral therapy aimed at transforming fear memories to treat PTSD. In sum, understanding how fear memory updates holds significant promise for PTSD treatment.

## A Synopsis of Post-Traumatic Stress Disorder (PTSD)

### PTSD

PTSD is a psychiatric condition characterized by intense fear and memories stemming from past traumatic experiences. It affects ~3.9% of the global population over their lifetime, with rates rising to 5.6% among trauma victims [1] and exceeding 15% among global pandemic survivors [2]. Women are twice as susceptible as men [3]. The coronavirus disease 2019 (COVID-19) pandemic has exacerbated PTSD symptoms worldwide [4, 5], highlighting significant challenges for existing mental health systems [6, 7]. Exposure therapy (ET) is a first-line treatment that facilitates fear memory extinction by safely exposing individuals to traumatic memories [8, 9]. However, this psychotherapy fails to expel fear memories permanently, leading to recurrence and treatment non-completion [10–13]. Given the substantial increase in its prevalence following the COVID-19 pandemic [4, 5], effective management of PTSD, which requires extensive time and professional management, poses significant challenges [11]. Thus, developing more efficient therapeutic strategies urgently requires a deeper understanding of fear memory mechanisms and effective interventions to alleviate the symptoms of PTSD.

**Fig. 1:**
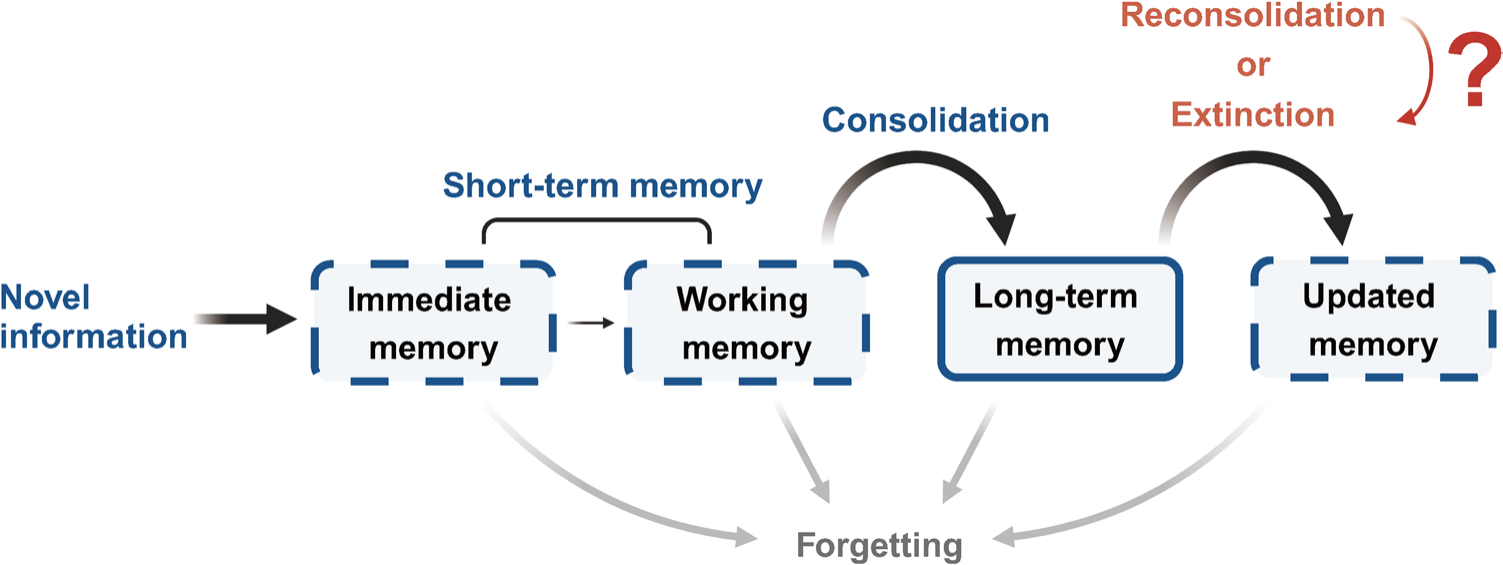
A schematic overview of memory simply includes short-term memory (STM), long-term memory (LTM), and updated memory. When new information is learned, it initially forms immediate memory, which lasts for a few minutes and can be extended through review, turning into working memory. Both immediate- and working-memory are termed STM since they are transient. LTM is established through consolidation, making memories more resistant to alterations. Processes like reconsolidation or extinction can update LTM by modifying original memories with new associations. Memories eventually fade if not reinforced. Unstable forms of memories are presented with dashed lines

Memory is a dynamic neurophysiological process that retrieves learned information through associative mechanisms [14, 15]. It is widely accepted to involve several stages: encoding, storing, recalling, and forgetting [15] (Fig. 1). Initially, newly-acquired information is encoded rapidly, with immediate memory fading quickly without rehearsal. Reviewing this information helps stabilize memory temporarily against external interference. Short-term memory, crucial for establishing long-term memory, lasts minutes to hours and encompasses immediate and working memory [16]. The process by which newly-encoded memories stabilize over time is termed “consolidation” [17]. As memory consolidates, it becomes resistant to disruption, transforming into long-term memory, which can persist for months [18, 19]. It was broadly agreed that memory consolidation followed a linear progression [20–22] until Prof. Nader introduced the concept of “reconsolidation”, where memories can be reactivated and updated without re-exposure to the original learning environment [23]. Re-exposure to a conditioned stimulus (CS) can either reinforce fear memory retention or facilitate extinction, where a previously unsafe context no longer provokes a fear response [24, 25]. Ideally, memories should be preserved at a level that retains knowledge of threats without negatively affecting mental well-being [26, 27]. Failure to achieve this balance may lead to anxiety and other psychiatric disorders, including PTSD [28, 29]. Current psychotherapies of PTSD often focus on deactivating or impairing strong emotional memories, such as traumatic memories [30, 31]. Recent research on fear memory extinction within the reconsolidation window, known as the “retrieval-extinction protocol”, shows promise in rapidly eliminating fear memories [32, 33]. However, mixed results highlight challenges in achieving consistent outcomes [34–37]. The balance between reconsolidation and extinction processes during memory retrieval is unpredictable [38].

In this review, we discuss the fundamental aspects of memory reconsolidation and extinction, focusing on the retrieval-extinction protocol and its potential clinical applications. We highlight its relevance for understanding memory modification and its implications for targeting maladaptive memories, particularly those associated with fear and trauma-related disorders [39, 40].

### Experimental Models of Fear Conditioning

**Fig. 2:**
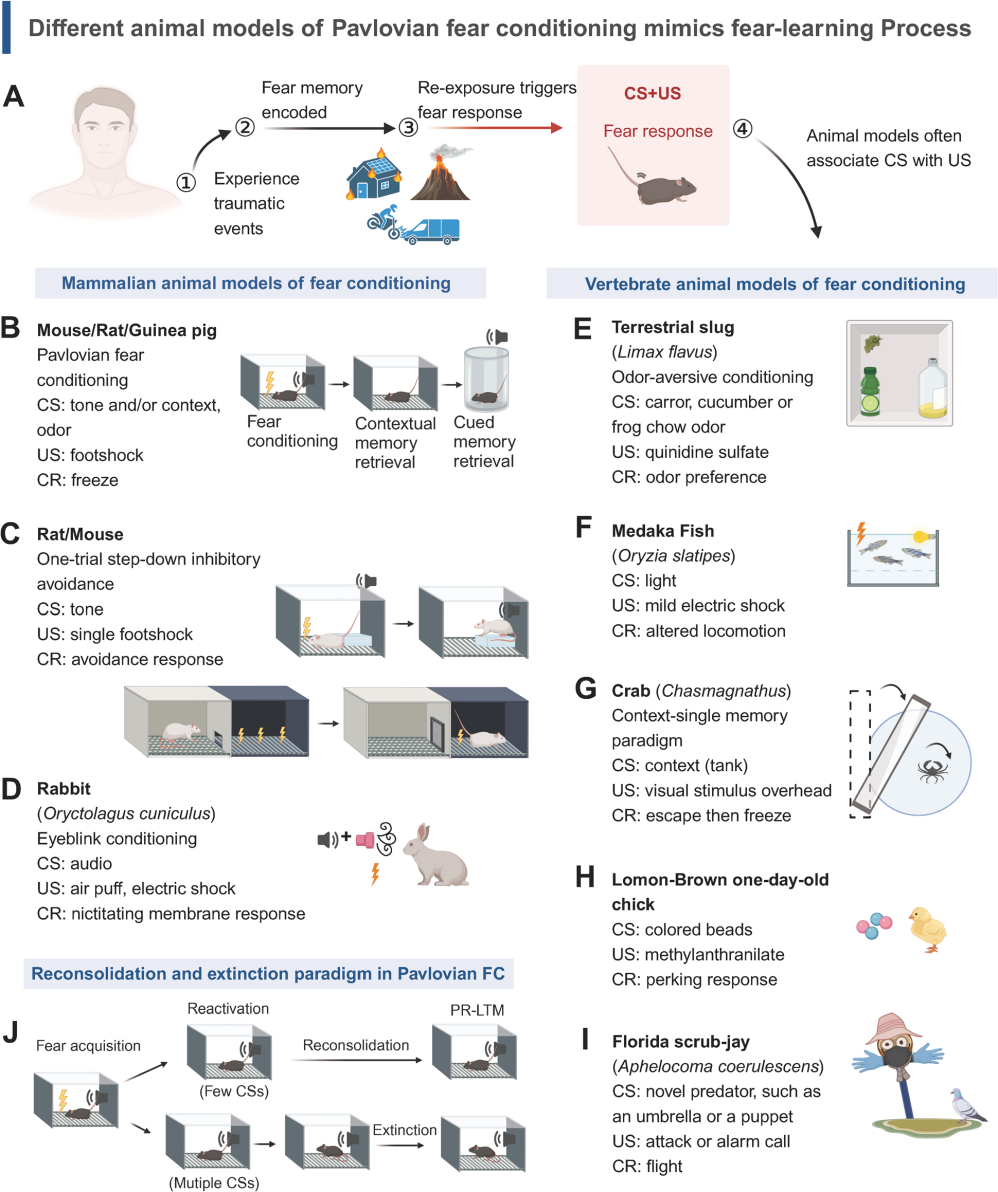
Diagrams of experimental animal models for Pavlovian fear conditioning. **A** Traumatic memories, such as experiencing a fire, a volcano erupting, or a car accident, are stored and retrieved upon re-exposure, forming associative learning processes studied in animal models. These experiments combine CS and US to induce fear responses in various mammalian and vertebrate subjects. **B-I** Different animal models of fear conditioning. **J** An overview of memory reconsolidation and extinction paradigms in the Pavlovian FC paradigm. After the fear acquisition, rodents are re-exposed to CSs in the training context to initiate either memory reconsolidation or extinction retrieval. They are then tested for memory retention in post-reactivation long-term tests (often performed 24 h after reactivation)

To understand the mechanisms of memory reconsolidation and extinction, we study them in experimental paradigms. Scientists have developed classical paradigms to study learning and memory, the classical conditioning pioneered by Ivan Pavlov being foundational [16, 41] (Fig. 2). Pavlov paired a neutral stimulus (CS), like a bell, with an unconditioned stimulus (US), such as food, to elicit a conditioned response (CR), like salivation. This laid the groundwork for studying neuronal mechanisms linking two events, predicting CS outcomes based on the US in specific contexts.

Classical fear conditioning, also known as Pavlovian fear conditioning (FC), pairs a CS (e.g., tone, odor, context) with a US (e.g., electric shock), to elicit a fear response [42]. In rodents, fear is shown through freezing in response to unexpected shocks, and memory is assessed by freezing duration during conditioning trials (Fig. 2B). Auditory or contextual fear conditioning (CFC) uses tone or context as the CS paired with the US to study different fear memory forms [43–47]. The one-trial step-down inhibitory avoidance (IA) paradigm measures fear-motivated learning by having rodents avoid electric shocks on a grid floor, with step-down latencies indicating IA memory retention [48–50]. Another paradigm, step-through avoidance (Fig. 2C), tests fear memory by measuring the latency to enter a dark box after experiencing shocks [51]. In the context-single memory (CSM) paradigm, crabs are put in a training context and exposed to an overhead visual danger stimulus (VDS), driving them to escape from the visual cue (Fig. 2G). With repeated VDS presentations, crabs develop a strong freezing response instead of escaping [52]. Successful training paradigms enable subjects to associate cues with aversive events, assessed by the degree of freezing, startle reflex, or locomotor activity [53, 54]. Classical fear conditioning has been widely studied across species, from mammals to birds and invertebrates (Fig. 2B-I) [55–61].

In studies of memory reconsolidation and extinction, researchers apply various manipulations during memory retrieval to induce different memory outcomes (Fig. 2J). In Pavlovian FC, the number of CSs presented during retrieval determines whether memory reconsolidation or extinction occurs [62–65]. CFC involves reactivating fear memories in the original or novel context to elicit reconsolidation or extinction, respectively [66, 67]. Some studies reintroduce animals into a novel context for a short duration each day, but this exposure lasts for several consecutive days [68, 69]. Other experiments also vary the number of CSs during retrieval to define reconsolidation or extinction [64–70]. Similarly, in the IA paradigm, subjects are exposed to the training chamber for distinct lengths of time to define two memory forms [71].

## Introduction of Reconsolidation and Extinction

This review aims to discuss the retrieval-extinction protocol as an alternative therapy for PTSD treatment. To begin with, we explore the origins and mechanisms underlying memory reconsolidation and extinction, with a focus on their neuronal and molecular profiles. Examining the interplay between these processes provides insights into how memories are modified and regulated over time. Specifically, we explore the neuronal and molecular mechanisms involved in reconsolidation and extinction, integrating experimental evidence from animal models and human fMRI. In this aspect, we seek to contribute to a deeper understanding of memory dynamics and their implications for therapeutic interventions.

### Memory Reconsolidation: Origins, Mechanisms, and fMRI Insights

Over a century ago, Müller introduced the term “Konsolidierung” to describe the phase when memories become resistant to interference [17]. They found that interventions following training could impair memory recall, while subsequent recall improved retrieval. Later, Misanin’s research discovered that delivering an electroconvulsive shock (ECS) to fear-conditioned rats after CS exposure induces amnesia [20]. His findings revealed the concept of “reconsolidation”. Reconsolidation, unlike consolidation, is a dynamic process that updates retrieved memories with new protein synthesis. Studies have shown that reconsolidation occurs more quickly and is more susceptible to amnesia than consolidation [72], emphasizing its role in memory modification and maintenance.

Reconsolidation challenges the traditional view of memories as static and unchangeable, suggesting that they are dynamic and can be modified. It occurs during memory retrieval, stabilizing recalled memories in a time-sensitive manner, which is crucial for interventions aimed at reducing fear of memories. Research shows that amnesic agents can disrupt reactivated memories when administered 6 h post-retrieval [73], while treatments before or up to 4 h after retrieval leave fear responses intact [18, 74]. This highlights a reconsolidation window, typically between 0.5 and 6 h post-retrieval, during which memories are more susceptible to modification [75]. Moreover, interventions applied strategically during reconsolidation have been shown to improve retention performance [76, 77]. These findings are key for developing PTSD therapies that target existing memories during this malleable phase.

**Fig. 3:**
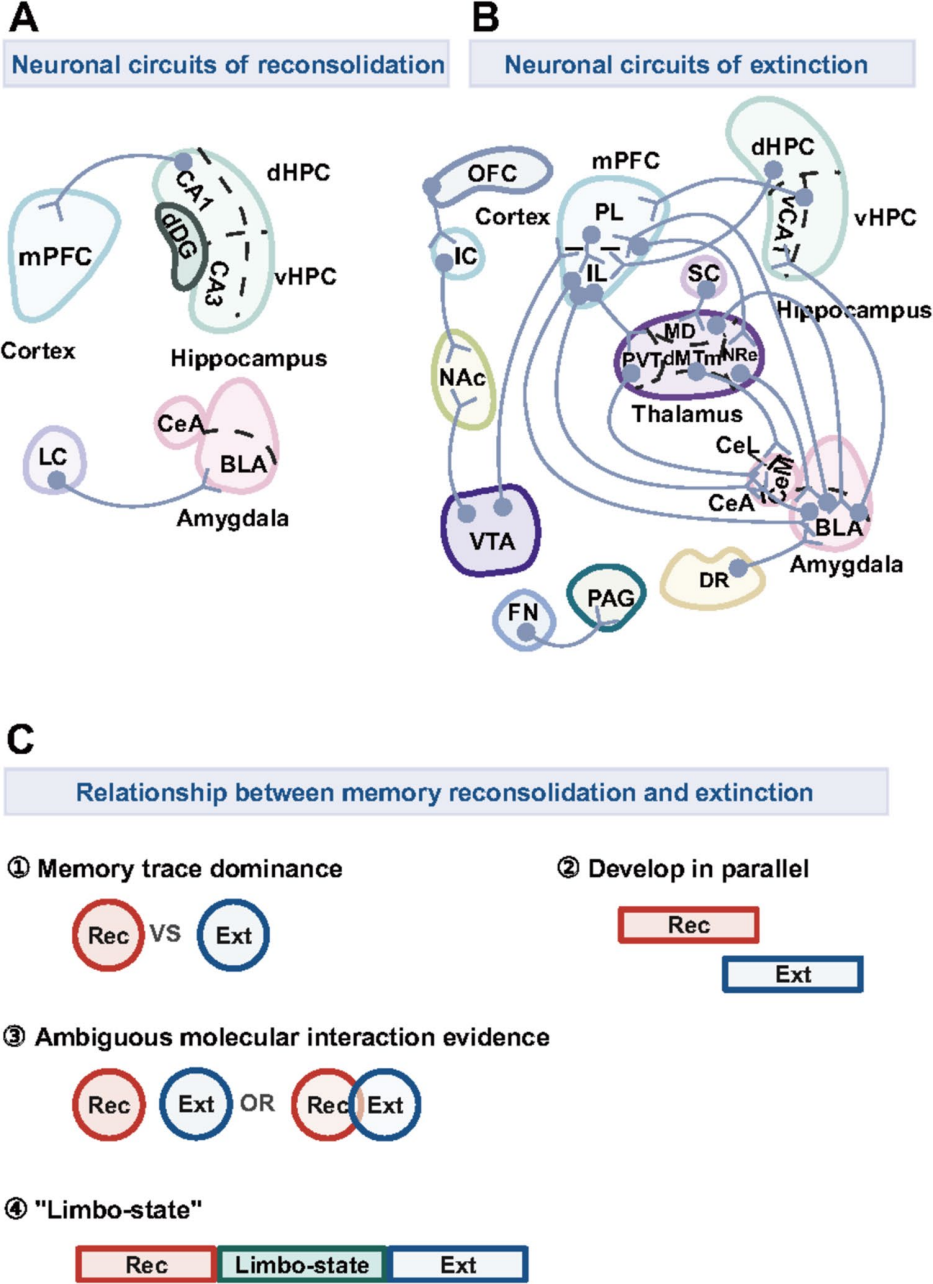
Conceptual diagrams of the neuronal circuits of reconsolidation and extinction, as well as their relationship. **A** Neuronal circuits of reconsolidation mainly involve the cortex, amygdala, and hippocampus. **B** Neuronal circuits of extinction form a cooperative network across the brain. **C** (1). Memory trace dominance theory implies a competitive mechanism between reconsolidation and extinction. (2). Nader et al. and Pérez-Cuesta et al. indicate that extinction does not prevent reconsolidation, suggesting dual memory periods may develop in a counterbalanced manner. (3). Molecular evidence regarding reconsolidation and extinction remains unclear from previous studies. (4). The linear memory assumption posits a distinct and resilient period between reconsolidation and extinction of memories. (Rec, Reconsolidation; Ext, Extinction.)

Understanding how different brain areas interact during memory reconsolidation is crucial for developing therapeutic approaches to disorders like PTSD, where eliminating the fear of memories is key. Several brain regions, particularly the amygdala and hippocampus, play central roles in memory updating and reconsolidation by reorganizing neuronal circuits [78], as shown in Table 1. The amygdala has been extensively studied for its role in the encoding, expression, extinction, and updating of fear memories. Its central region (CeA) plays a key role in encoding salient-specific valence [79]. In addition, the basolateral nucleus of the amygdala (BLA) is responsible not only for processing both positive and negative stimuli [80] but also for storing memory [81]. Despite extensive investigations using lesion and pharmacological approaches, the neuronal mechanisms underlying the amygdala’s involvement in reconsolidation remain inadequately understood. To illustrate, pharmacological inhibition of Thy1-expressing neurons in the BLA [51], or disrupting noradrenergic projections from the locus coeruleus to the BLA can block fear memory reconsolidation [82]. Correspondingly, the hippocampus, crucial for episodic memory and emotional behaviors [83–85], also plays a significant role in reconsolidation [86]. Memory destabilization reduces neuronal activity in regions like CA1, and CA3, and optogenetic inhibition of CA1 during retrieval can cause memory failure [87]. Blocking the projection from the dorsal hippocampus to prelimbic regions of the medial prefrontal cortex (dHPC-mPFC) before retrieval also prevents reconsolidation [88]. Interestingly, identical neuronal populations across distinct brain regions can produce opposing effects. Optogenetic activation of the dorsal dentate gyrus (dDG) during recall blocks memory reconsolidation [66], whereas pharmacological inhibition of the lateral neocortex before re-exposure to the CS achieves a similar result [89]. This discrepancy suggests that diverse populations of neurons, such as CaMKII+ neurons (Ca^2+^-calmodulin-dependent protein kinase II-positive neurons), play various roles in CFC. However, research on neuronal circuits and the roles of specific neuron types during reconsolidation remains limited (Fig. 3A). Thus, there is a critical need for further studies to prioritize and explore this area.

**Table 1:** Molecular mechanisms of memory reconsolidation

Paradigm	Species	Brain areas	Drug administration				Effect		References
			Target	Classification	Drug	Time	Within-session recon.	Recon. retention	
Pavlovian FC	Sprague-Dawley rat	Systemic	Protein synthesis	Inhibitor	ANI	Post-retrieval	↓	↓	[193]
	Wistar rat				CXM		↓	↓	[194]
	Sprague-Dawley rat	ACC	Protein synthesis	Inhibitor	ANI	Post-retrieval	↓	↓	[195]
		LA			CXM		–	↓	[196]
	Lister rat	Systemic	β-adrenergic receptor	Antagonist	Propranolol	Post-retrieval	–	↓	[197]
		Amygdala					–	↓	
	Sprague-Dawley rat	Systemic	NMDA-R	Partial agonist	DCS	Post-retrieval	–	↓	[198]
		Amygdala					–	↑	
		Systemic		Noncompetitive receptor antagonist	MK-801	Pre-retrieval	–	↑	
	Wistar rat	Systemic	Glucocorticoid receptor	Antagonist	RU38486	Post-retrieval	No effect	↓	[199]
	Sprague-Dawley rat	BLA			RU486		–	↓	[74]
						6 h post-retrieval	–	No effect	
		LA	AMPA-R	Blocker	NASPM	Post-retrieval	–	↓	[200]
			ERK-MARK signaling pathway	Inhibitor	U0126	Post-retrieval	–	↓	[196]
	C57BL/6 mouse	Systemic	MEK signaling pathway	Inhibitor	SL327	Post-retrieval	–	↓	[201]
	ERK1 mutant mouse								
		BLA	Protein kinase A	Activator	6-BNZ-cAMP	Post-retrieval	–	↑	[202]
				Inhibitor	Rp-cAMPS		–	↓	
	C57BL/6 mouse	Systemic	mTOR	Selective inhibitor of mTOR kinase	Rapamycin	Post-retrieval	↓	↓	[203]
						12 h post-retrieval	↓	–	
						24 h post-retrieval	No effect	–	
	Sprague-Dawley rat	BLA	NF-κB signaling pathway	Direct inhibitor of NF-κB DNA-binding complex	SN50	Pre-retrieval	No effect	↓	[204]
				IKK inhibitor	SSZ	Post-retrieval	No effect	↓	
		CeA		IKK inhibitor	SSZ		No effect	No effect	
		BLA	Rac	Inhibitor	NSC23766	Post-retrieval	↓	–	[205]
		CeA					No effect	–	
		CA1					No effect	–	
	C57BL/6 mouse	Systemic	HDAC	Inhibitor	VPA	90 s pre-retrieval	No effect	↑	[76]
	Sprague-Dawley rat	LA	HDAC	Inhibitor	TSA	1 h post-retrieval	↑	–	[77]
			DNMT	Inhibitor	5-AZA		↓	–	
					RG108		↓	–	
			mRNA synthesis	Inhibitor	α-Amanitin	Post-retrieval	No effect	↓	[206]
			Arc/Arg3.1 early gene	Antisense	ODNs	1 d post-conditioning	No effect	↓	[207]
	C57BL/6 mouse	BLA	Actin filament	Arrest	Phalloidin	0.5 h post-retrieval	↓	–	[75]
						6 h post-retrieval	↓	–	
						24 h post-retrieval	No effect	–	
CFC	C57BL/6 mouse	Systemic	Protein synthesis	Inhibitor	ANI	Post-retrieval	↓	–	[208]
		dHPC					↓	–	
		ACC					–	–	
	Wistar rat	CA1					–	↓	[209]
	Long-Evans rat	Amygdala	Protein synthesis	Inhibitor	ANI	Post-retrieval	–	↓	[210]
	Wistar/ST rat	Systemic	NMDA-R	Partial agonist	DCS	Post-retrieval	–	↑	[211]
	C57BL/6 mouse	Systemic		Noncompetitive receptor antagonist	MK-801	Post-retrieval	–	↓	[212]
	Lister rat	dHPC		Antagonist	D-AP5	Post-retrieval	↓	↓	[213]
	C57BL/6 mouse	Systemic	AMPA-R	Potentiator	PEPA	Pre-retrieval	No effect	No effect	[214]
		BLA							
	Wistar rat	NR	N2A-containing receptor	Antagonist	TCN-201	Post-retrieval	–	↓	[215]
		Systemic	CB1/2 receptor	Inhibitor	WIN55,212-2	Post-retrieval	No effect	–	[209]
		Amygdala	CB1 receptor	Antagonist	AM251	Post-retrieval	–	↓	[216]
		CA1					↑	↑	[217]
			CB1 receptor	Antagonist	AEA		↓	–	
		Systemic	GABA_A_ receptor	Agonist	MDZ	Post-retrieval	–	↓	[218]
	Sprague-Dawley rat	Systemic	GABA_A_ receptor	Partial agonist	Flumazenil	Post-retrieval	–	↓	[219]
	Wistar rat	NR	GABA_A_ receptor	Agonist	Muscimol	Post-retrieval	–	↓	[215]
	C57BL/6 mouse	Systemic	H3 receptor	Inverse agonist	Thioperamide	Post-retrieval	–	No effect	[212]
	Female C57BL/6 mouse				Pitolisant	Post-retrieval	No effect	–	[220]
	Wistar rat	Amygdala	H3 receptor	Antagonist	Thioperamide	Post-retrieval	–	–	[216]
		CA1	5-HT_5A_ receptor	Antagonist	SB-699551	Post-retrieval	–	No effect	[209]
						3 h post-retrieval	–	↓	
			5-HT_6_ receptor	Antagonist	SB-271046	Post-retrieval	–	No effect	
						3 h post-retrieval	–	No effect	
				Agonist	WAY-208466	Post-retrieval	–	No effect	
						3 h post-retrieval	–	↓	
			5-HT_7_ receptor	Antagonist	SB-269970	Post-retrieval	–	No effect	
						3 h post-retrieval	–	↑	
				Agonist	AS-19	Post-retrieval	–	No effect	
						3 h post-retrieval	–	No effect	
		Amygdala	Muscarinic receptor	Antagonist	Scopolamine	Post-retrieval	–	–	[216]
	Sprague-Dawley rat	Systemic	NF-κB signaling pathway	Inhibitor	DDTC	Post-retrieval	↓	↓	[221]
	Lister rat	dHPC	MEK-ERK cascade pathway	Inhibitor	SSZ	Pre-retrieval	↓	↓	[213]
			IKK-NF-κB signaling pathway	Inhibitor	U0126		No effect	No effect	
		CA1	IL-1 receptor signaling pathway	Endogenous IL-1 antagonist	IL-1ra	Post-retrieval	↓	↓	[222]
	Long-Evans rat	Amygdala	CaMKII signaling pathway	Inhibitor	Myr-AIP	Post-retrieval	No effect	↑	[95]
	C57BL/6 mouse	dHPC	AMPAR endocytosis	Blocker	TAT-GluA23Y	1 h pre-retrieval	↑	-	[223]
						15 min post-retrieval	↑	–	
						1 d post-retrieval	↑	–	
	Wistar rat	PL	Protein kinase C activity	Estrogen receptor modulator	Tamoxifen	Post-retrieval	↓	↓	[67]
						6 h post-retrieval	No effect	↓	
		ACC				Post-retrieval	No effect	↓	
						6 h post-retrieval	No effect	↓	
		PL	PKMζ inhibition	Inhibitor	Chelerythrine	Post-retrieval	↓	↓	[224]
					ZIP	1 h post-retrieval	↓	↓	
	Sprague-Dawley rat	BLA	Rac	Inhibitor	NSC23766	Post-retrieval	No effect	–	[205]
		CeA					No effect	–	
		CA1					↓	–	
	Long-Evans rat	Amygdala	mRNA	Inhibitor	ACT-D	Post-retrieval	No effect	No effect	[210]
	C57BL/6 mouse	Systemic	mTOR	Selective inhibitor of mTOR kinase	Rapamycin	Post-retrieval	↓	↓	[225]
		BLA	Actin filament	Arrest	Phalloidin	0.5 h post-retrieval	↓	–	[75]
						6 h post-retrieval	No effect	–	
						24 h post-retrieval	No effect	–	
	Wistar rat	BLA	Sodium channel	Blocker	TTX	Post-retrieval	–	↓	[216]
						96 h post-retrieval	↓	–	[226]
		NBM					No effect	–	
		ENT					↓	↓	[227]
		Systemic	Ca^2+^ signaling pathway	Inhibitor	PD150606	Post-retrieval	–	↓	[228]
	C57BL/6 mouse	dHPC		Inhibitor	ALLN	Post-retrieval	–	↓	[229]
	Lister rat	dHPC	Zif268	ASO	Zif268-ODN	Pre-retrieval	↓	↓	[222]
			BDNF		BDNF-ODN	1.5 h pre-training	↓	↓	
Sequential FC	C57BL/6 mouse	Systemic	β1-adrenergic receptor	Antagonist	Propranolol	Post-US retrieval	No effect	↓	[96]
					Betaxolol		–	↓	
			NMDA	Antagonist	APV	Post-US retrieval	–	↑	
IA	Wistar rat	Systemic	Glucocorticoid receptor	Antagonist	RU38486	Post-retrieval	↓	↓	[199]
		Hippocampus					↓	↓	
	Sprague-Dawley rat	Systemic	mTOR	Inhibitor	Rapamycin	Post-retrieval	–	↓	[230]
		PL					–	↓	
		IL					–	↑	

Recon, reconsolidation; ACC, anterior cingulate cortex; IL, infralimbic region of medial prefrontal cortex; LA, lateral amygdala; CeA, central amygdala; CA1, a subregion of hippocampus; NR, nucleus reuniens; NBM, nucleus basalis magnocellularis; ENT, entorhinal cortex; NMDA-R, N-methyl-D-aspartic acid receptor; AMPA-R, α-amino-3-hydroxy-5-methyl-4-isoxazolepropionic acid receptor; BDNF, brain-derived neurotrophic factor; ERK-MAPK signaling pathway, extracellular signal-regulated kinase (ERK)—mitogen-activated protein kinase (MAPK) signaling pathway; MEK signaling pathway, mitogen-activated protein kinase signaling pathway, mTOR, mechanistic target of rapamycin signaling pathway; NF-κB signaling pathway, nuclear factor kappa-B signaling pathway; IKK-NF-κB signaling pathway, IκB kinase-nuclear factor kappa-B signaling pathway; IL-1, interleukin-1; CaMKII, Ca^2+^-calmodulin (CaM)-dependent protein kinase II; PKMζ, protein kinase M zeta; ASO, antisense oligonucleotides; ACT-D, actinomycin-D; AEA, anandamide; ALLN, N-Acetyl-Leu-Leu-norleucinal; AM251, N-(piperidin-1-yl)-5-(4-iodophenyl)-1-(2, 4-dichlorophenyl)-4-methyl-1H-pyrazole-3-carboxamide; ANI, anisomycin; APV, aminophosphonovaleric acid; CXM, cycloheximide; D-AP5, D-2-Amino-5-phosphovaleric acid; DCS, D-Cycloserine; DDTC, diethyldithiocarbamate; DRB, 5,6-dichloro-1-b-d-ribofuranosylbenzimidazole; MDZ, midazolam; MK-801, dizocilpine; myr-AIP, myristoylated autocamtide-2 related inhibitory peptide; NASPM, 1-naphthylacetylsperimine; ODNs, antisense oligodeoxynucleotides; PEPA, 4-[2-(phenylsulfonylamino)ethylthio]-2,6-difluorophenoxyacetamide; Rac, Ras-related C3 botulinum toxin substrate; Rp-cAMPS, Rp-adenosine 3’,5’-cyclic monophosphorothioate; RG108, N-Phthalyl-L-Tryptophan; SSZ, sulfasalazine; TAT-GluA23Y, HIV TAT-fused GluA2-derived C-terminal peptide; TCN-201, fluoro-N-[4-[[2-(phenylcarbonyl)hydrazino]carbonyl]benzyl]benzenesulphonamide; TSA, trichostatin A; TTX, tetrodotoxin; VPA, valproic acid; ZIP, zeta inhibitory peptide; 5-AZA, 5-AZA-2’-deoxycytidine; 5-HT, serotonin; 6-BNZ-cAMP, N6-benzoyladenosine-3’,5’-cyclic monophosphate

When fear memories are retrieved, increased expression of immediate-early genes like *Zif268*, *Arc*, and *c-Fos* is evident in key brain regions, such as the hippocampus, the amygdala, and the nucleus accumbens (NAc) [90–93]. Genetic studies have shown that serum- and glucocorticoid-induced kinase 3 expression is elevated following retrieval and phosphorylation of GluA1 at Ser831 and Ser845 in the amygdala, hippocampus, and mPFC [94]. Activation markers of proteasome-dependent protein degradation, such as the Lys48-linked poly-ubiquitin chain, also increase following fear memory retrieval [50]. Reactivated fear memories also induce the phosphorylation of CREB and Rpt6-S120, and upregulate proteasome activity in the amygdala and hippocampus [91, 95, 96]. Research on memory reconsolidation often applies pharmacological manipulation targeting diverse receptors or signaling pathways by specific inhibitors or agonists to disrupt the reactivated memory phase. It possesses unique molecular profiles across brain regions, such as CREB-mediated transcription, intracellular signaling pathways, neurotransmitters, or some second messengers (Table 1). For further details, refer to high-profile reviews in the field [97].

Consistent with evidence from rodents, functional magnetic resonance imaging (fMRI) in both healthy subjects and traumatized patients establishes that reconsolidation also requires the cooperation of multiple cerebral areas, notably the amygdala and hippocampus (Table 2). Increasing amygdaloid activity during fear memory retrieval suggests that it predicts fear expression and the activation of fear-related circuits [39]. The hippocampus is responsible for the modification of fear memories during re-exposure, particularly responding to unpredicted reminders associated with the original fear learning context [98]. In declarative tasks, hippocampal activity significantly increases when subjects encounter unpredictable syllable cues, aligning with patterns found during memory reactivation. fMRI studies also highlight a coactivated network involving the midline anterior cingulate cortex (ACC), insular cortex (IC), or amygdala-hippocampus circuit, when addressing dysfunctional fear memories [39]. Enhanced activity in regions like the IC, thalamus, parahippocampal gyrus, and mid-frontal gyri potentially contribute to alleviating the overwhelming stress in traumatized patients following treatments that impair reconsolidation, such as propranolol-induced interventions [99].

**Table 2:** Human fMRI evidence of memory reconsolidation

Paradigm	Subjects	Anatomical structure	Hemisphere	Test memory period	Activity	Function	References
Fear conditioning	Healthy subjects	Basolateral amygdala	Bilateral	1 d post 10 min post-Rec. +Ext.	>	Predict the return of fear	[39]
				1 d post 6 h post-Rec. +Ext.	<		
		dACC	Bilateral	During react.	<		[128]
		vmPFC			<		
		vmPFC-amygdala			<	Erase fear memory	
		dlPFC	Bilateral	Post-react.	>	Reduce fear	[40]
Declarative task	Healthy subjects	Hippocampus	Bilateral	Early retrieval post-rec.	<	–	[231]
				Late retrieval post-rec.	<		
		Parahippocampus		Early retrieval post-rec.	<		
				Late retrieval post-rec.	<		
		Prefrontal cortex		Early retrieval post-rec.	<		
				Late retrieval post-rec.	<		
		Parietal lobe		Early retrieval post-rec.	<		
				Late retrieval post-rec.	<		
		Temporal lobe		Early retrieval post-rec.	<		
				Late retrieval post-rec.	<		
		Posterior cingulate cortex		Early retrieval post-rec.	>		
				Late retrieval post-rec.	<		
		Anterior cingulate cortex		Early retrieval post-rec.	>		
				Late retrieval post-rec.	<		
		Hippocampus	Left	During React.	<	Detect incongruence and update memory	[98]
Over faces task	PTSD patients	Thalamus	Right	Propranolol-induced reconsolidation impairment	>	–	[99]
		ACC	Right		<	Decrease PTSD symptom	
		Mid-frontal gyri	Bilateral		<		
		Mid-cingulate	Bilateral		<		
Face-location association learning paradigm	Healthy subjects	Hippocampus	Left	After new learning-induced during Rec.	<	Process declarative memories	[232]
		Amygdala	Right		<		
Two-day exposure protocol	Arachnophobia subjects	Amygdala	Bilateral	10 min exposure prior to reactions.	>	Predict fear	[167]
Three-day retrieval extinction protocol	Healthy subjects	IT	Bilateral	Post-retrieval extinction	>	Prediction error	[137]
		dlPFC			>		
		dlPFC-ACC			>		
Autobiographical memory procedure	Healthy subjects	Hippocampus	Bilateral	During react.	<	Positive information update	[233]
		Ventral striatum			<		

Rec, Reconsolidation; Ext., Extinction; React., Reactivation; <, increased activity; >, decreased activity; ACC, anterior cingulate cortex; dACC, dorsal anterior cingulate; vmPFC, ventromedial prefrontal cortex; dlPFC, dorsolateral prefrontal cortex; IT, inferior temporal cortex

### Memory Extinction: Origins, Mechanisms, and fMRI Insights

The study of extinction tracks back to Pavlov’s classic experiment where he recorded the gradual disappearance of a dog’s salivary response to food cues when those cues were presented without food [41, 100]. This process, known as “extinction”, refers to the gradual weakening of a CR [101]. Unlike forgetting, extinction involves the formation of a new memory that associates a CS with the absence of a US, requiring new protein synthesis for memory maintenance [102, 103]. Experimental approaches to extinction involve repeated exposure to a CS alone or prolonged exposure in a conditioning context to reduce CRs. The prediction error (PE) between expected and actual outcomes destabilizes the original memory, facilitating the formation of a new CS-no US memory that competes with the original CS-US memory. Extinction encompasses acquisition, retrieval, and retention periods, but over time, vulnerabilities like spontaneous recovery can lead to the return of the original fear memory [104]. Context renewal occurs when a CR reappears outside the extinction context, and reinstatement refers to the return of fear responses if the US unexpectedly reappears, reinstating the CS-US association [105].

The cortex, amygdala, and hippocampus collaborate in fear extinction memory, reflecting an emerging interest in their role in whole-brain networks [28]. Since the neuronal circuits of extinction memory have been well documented [106], in this review we outline neuronal circuits involving diverse subpopulations, summarizing various manipulation techniques such as pharmacological interventions, optogenetics, Tetanus toxin light chain, and optogenetically-induced long-term potentiation across extinction stages (Fig. 3B and Table 3).

**Table 3:** Summary of neuronal circuits of extinction memory in fear conditioning

Paradigm	Extinction period	Neuronal circuits	Neuronal type	Manipulation	Effect on memory	Ref.
Pavlovian FC	Fear conditioning	OFC-IC-NAc	–	Optogenetic activation	↑ ext. retrieval	[69]
	Fear conditioning+ Ext. acquisition	IC-NAc	–	TetTox inactivation	↓ ext. retrieval	
	Pre-ext. acquisition	VTA-AcbSh	Dopamine	Pharmacological activation	↑ext. acquisition	[234]
		mPFC-RE	–	Pharmacological inhibition	↓ext.	[158]
		BLA-mPFC	CaMKII	Optogenetically induced-LTD	↑ext.	[114]
	Ext. acquisition	PL-IL	Pyramidal	Optogenetic activation	↑ext. acquisition	[111]
		IL-BLA	CaMKII	Optogenetic activation	↑ext.	[110]
		VTA-NAc mShell	Dopamine	Optogenetic inhibition	↓ext. consolidation	[235]
		VTA-vmPFC	Dopamine		↓ext. acquisition	
		DR-BA	5-HT	Optogenetic activation/inhibition	↓/↑ ext.	[236]
		FN-vlPAG	Glu→Glu+GABA	Optogenetic activation	↑ext.	[237]
		SC-MD	GABA→CaMKII	Optogenetic inhibition	↑ext.	[238]
		MD-BLA	CaMKII-pyramidal	Optogenetic activation	↓ext.	
		dMTm-CeA	CaMKII	Optogenetic inhibition	↑ext.	[239]
		BLA-vCA1	Glutamatergic	Optogenetic inhibition	↓ext.	[119]
	Pre-test	BLA-CeL	Glutamatergic	Pharmacological inhibition	↓ext.	[68]
	Pre-ext. retrieval	IL-PVT	Glutamatergic	Pharmacological inhibition	↓ext. retrieval	[240]
		PVT-CeL				
	Ext. retrieval	dHPC-IL	CaMKII	Optogenetic activation/inhibition	↑/↓ext. retrieval	[118]
		BLA-mPFC	–	Optogenetic modulation	↑ext. retrieval	[115]
		vHPC-mPFC				
	Ext. renewal	vHPC-PL	Glu→GABA	Pharmacological inhibition	↑ext.	[117]
CFC	Ext. acquisition	PL-amygdala	Pyramidal	Optogenetic activation	↓ext.	[109]
	Ext. retrieval	NRe-BLA	–	Optogenetic activation/inhibition	↑/↓remote ext.	[112]

Ext., extinction; AcbSh, nucleus accumbens shell; BA, basal amygdala; CeL, central amygdala; dMTm, dorsal midline thalamus; DR, dorsal raphe nucleus; FN, fastigial nucleus; MD, mediodorsal thalamus; NAc mShell, medial shell of nucleus accumbens; NRe, thalamic nucleus reuniens; RE, thalamic nucleus reuniens; OFC, orbitofrontal cortex; PVT, paraventricular nucleus of the thalamus; SC, superior colliculus; vlPAG, ventrolateral periaqueductal gray; vmPFC, ventromedial prefrontal cortex; VTA, ventral tegmental area; GABA, gamma-aminobutyric acid; Glu, glutamate

The PFC plays a key role in the formation and retrieval of extinction memory [107, 108]. Pyramidal projections from the prelimbic cortex (PL) to the amygdala regulate fear expression [109], while infralimbic cortex (IL)-BLA connections facilitate extinction acquisition [110]. The PL also sends excitatory afferents to the IL, which promotes extinction learning, particularly during early acquisition [111]. However, these conclusions lack verification of the tripartite neural circuit projections (PL-IL-BLA) regulating extinction learning. Pharmacological inhibition of the excitatory afferents from the PL to the paraventricular thalamus (PVT) or the PVT to the central lateral amygdala (CeL) before retrieval impairs extinction memory. Monosynaptic retrolabeling can be used to establish the role of the PL-PVT-CeL circuit in fear extinction. Interestingly, the thalamic nucleus reuniens (NRe) works as a hub for IL-BLA projections during remote extinction memory, as indicated by significant *c-Fos*+ activated cells in the IL-NRe and NRe-BLA projections. The anatomical circuit, verified by the retrograde virus method, suggests that a cortical-thalamic-amygdalar circuit plays a pivotal role in remote memory extinction [112]. Another brain structure that deserves further attention is the IC, a gateway to fear or extinction memory through projections to the CeA or NAc, respectively. The orbitofrontal cortex (OFC) exerts top-down control over this circuit; the OFC-IC-NAc pathway promotes extinction learning and expression [69].

The amygdala plays an essential role in memory extinction. Optogenetic activation of the Thy1-expressing subpopulation of glutamatergic BLA neurons during the extinction acquisition period promotes new learning and consolidation of extinction memory [113]. Similarly, optogenetic modulation of BLA-mPFC afferents improves extinction learning and retrieval both in the Pavlovian and contextual FC paradigms [114, 115]. This effect may be due to optogenetically-induced long-term depression, which reduces presynaptic excitability and fear responses during the extinction retention period. Fear conditioning alters corticotropin-releasing factor-expressing (CRF+) neurons in the CeA to non-CRF+ and somatostatin-expressing neurons in the BLA, while extinction learning reverses this transformation [68]. This dynamic remodeling highlights the inevitable role of excitatory CRF+ neurons in fear activity, as their excitation facilitates extinction learning and their inhibition reinforces fear memory. Intriguingly, inhibitory clusters of intercalated (ITC) neurons located between the basolateral and central region of the amygdala also influence the fear state [64]. The dorsal (ITCdm) and ventral (ITCvm) clusters respond to fear- and extinction-state, respectively. Further, stimulation of ITCvm neurons evokes postsynaptic responses in the medial CeA (CeM), which projects densely to the ventrolateral periaqueductal grey (vlPAG), suggesting that ITCvm neurons reduce the fear response by inhibiting the CeM-vlPAG pathway. Conversely, the ITCdm-BLA-mPFC pathway inhibits extinction learning by suppressing ITCvm neurons. These conclusions require further validation in experimental rodents.

Extinction memory is context-dependent, regulated by hippocampal activity, which is indispensable to spatial representation and fear memory, including extinction learning and fear relapse [116]. Pharmacological inactivation of the ventral hippocampus (vHPC) to the IL pathway facilitates fear extinction [117]. *Ex vivo* whole-cell recordings have revealed that local parvalbumin-expressing interneurons from the BLA mediate fear renewal by feed-forward inhibition. Likewise, optogenetic activation of the pathway from the dorsal hippocampus (dHPC) to the IL enhances fear extinction memory, but this effect is abolished by conditional deletion of extracellular signal-regulated kinase 2 in the IL [118]. The neighboring hippocampal subregions, dHPC and vHPC, contribute differently to fear extinction memory. Yet, there is a controversy over the role of the BLA in vHPC projections in CS-evoked activity during extinction memory [119]. Optogenetic silencing of this circuit during extinction learning significantly impairs extinction memory by degrading local theta-oscillations and weakening the spatial encoding capacity of the vHPC in a glutamatergic transmission-dependent manner.

Various neurotransmitters are important in fear extinction memory [120, 121]. Classical neurotransmitters (for example, γ-aminobutyric acid, glutamate, dopamine, cannabinoids, glucocorticoids, and norepinephrine) play critical roles in fear extinction memory encoding, recall, and retention. Moreover, intracellular signaling pathways such as L-type voltage-gated Ca^2+^ channels (LVGCCs) and second messengers are essential for extinction memory storage. These mechanisms are summarized in Table 4.

**Table 4:** Molecular mechanisms of extinction memory

Paradigm	Species	Brain areas	Drug administration				Effect		References
			Target	Classification	Drug	Time	Within-session ext.	Ext. retention	
Pavlovian FC	Lister rat	Systemic	NMDA-R	Partial agonist	DCS	Pre-Ext. acquisition	–	–	[198]
		BLA					–	↑	
		Systemic	NMDA-R	Noncompetitive NMDA-R antagonist	MK-801		–	↓	
	Sprague-Dawley rat	Systemic	Noradrenergic β-receptor	Blocker	Propranolol	Post-conditioning	↑	No effect	[241]
	C57BL/6 mouse	Systemic	HDAC	Inhibitor	VPA	Pre-Ext. acquisition	↑	↑	[76]
	KCTD8/12 KO mice	Systemic	GABA_B_	Agonist	Baclofen	Pre-Ext. acquisition	No effect	No effect	[242]
CFC	Lister rat	Systemic	LVGCCs	Blocker	Nimodipine	Pre-Ext. acquisition	↑	–	[243]
	Wistar rat	CA1	CB1-R	Antagonist	AM251	Post-Ext. acquisition	No effect	↓	[217]
		NBM	Sodium channel	Blocker	TTX	96 h post-conditioning	No effect	–	[244]
		SN					No effect	–	
		BLA					No effect	–	
		NBM				2 h post-Ext. acquisition	–	No effect	
		SN					–	No effect	
		BLA					–	↓	
		ENT				Post-Ext. acquisition	↑	↑	[227]
	C57BL/6 mouse	Systemic	NMDA-R	Partial agonist	DCS	Pre-Ext. acquisition	↑	↑	[214]
			AMPA-R	Potentiator	PEPA	Pre-ext. acquisition			
			mTOR	Inhibitor	Rapamycin	Post-Ext. acquisition	↑	↑	[225]
		dHPC	Ca^2+^ signaling pathway	Inhibitor	ALLN	Post-Ext. acquisition	–	↓	[229]
IA	Sprague-Dawley rat	Systemic	mTOR	Inhibitor	Rapamycin	Post-Ext. acquisition	↑	↑	[230]
		PL					No effect	–	
		IL					No effect	–	
	Wistar rat	Systemic	GABA		Topiramate	Ext. acquisition	↑	–	[245]

Ext., extinction; IA, inhibitory avoidance; KCTD,K+ channel tetramerization domain; BLA, basolateral nucleus of amygdala; CA1,, a subregion of hippocampus; NBM, nucleus basalis magnocellularis; SN, substantia nigra; ENT, entorhinal cortex; dHPC, dorsal hippocampus; PL, prelimbic region of medial prefrontal cortex; IL, infralimbic region of medial prefrontal cortex; NMDA-R, N-methyl-D-aspartic acid receptor; DCS, D-Cycloserine; MK-801, dizocilpine; myr-AIP, myristoylated autocamtide-2 related inhibitory peptide; HDAC, histone deacetylase; GABA_B_, gamma-aminobutyric acid type B receptor; LVGCCs, L-type voltage-gated Ca^2+^ channels; VPA, valproic acid; CB1-R, cannabinoid type-1receptor; AM251, N-(piperidin-1-yl)-5-(4-iodophenyl)-1-(2, 4-dichlorophenyl)-4-methyl-1H-pyrazole-3-carboxamide; TTX, tetrodotoxin; PEPA, 4-[2-(phenylsulfonylamino)ethylthio]-2,6-difluorophenoxyacetamide; mTOR, mechanistic target of rapamycin (mTOR) signaling pathway; ALLN, N-Acetyl-Leu-Leu-norleucinal; GABA, gamma-aminobutyric acid

The temporal dynamics of extinction memory in the human brain are characterized by connectivity between key structures, including the amygdala, hippocampus, and cortex [122–125]. Researchers have focused on hyperactivated or hypoactivated areas during fear acquisition, extinction training, and extinction recall (Table 5). Healthy volunteers exposed to the Pavlovian FC paradigm show strong activation of the amygdala and its connectivity with cortical areas or the anterior hippocampus [126–129]. Even in focal and bilateral vmPFC-lesioned patients, amygdala connectivity remains elevated with aversive stimuli [130], highlighting the vmPFC-amygdala circuit’s role in predicting fear, supported by the finding that proximal threats by visual reality facilitate learned fear memory *via* suppressing the amygdala-cortical circuit during fear acquisition and extinction [131, 132]. During extinction training, hippocampus-amygdala connectivity increases [129], as the hippocampus distinguishes in the absence of stimuli [133], and the amygdala facilitates novel associations when information changes [126]. The IC and dACC are also active during extinction memory [134, 135], with hyperactivity reported in PTSD patients and healthy participants during extinction conditioning, acquisition, or recall [136]. The dACC activity is governed by the dorsolateral PFC (dlPFC) as seen in studies where healthy volunteers undergoing Pavlovian FC show inactive states in the inferior temporal cortex (IT) and dlPFC, along with suppressed IT-dlPFC and dlPFC-ACC connections during extinction retrieval [137]. As extinction progresses, the auditory association cortex remains active, even when the activity of the vmPFC and amygdala decreases [138]. Unpredicted outcomes in changing contexts activate the posterolateral cerebellum and frontopolar OFC [125, 139]. Fear acquisition inactivates the cerebellum, which shows reduced activity during extinction retrieval [125, 132].

**Table 5:** Human fMRI evidence of memory extinction

Paradigm	Subjects	Anatomical structure	Hemisphere	Test memory period	Activity	Function	Ref.
Fear conditioning and extinction paradigm	Healthy subjects	Amygdala	Bilateral	Fear acquisition	<	Fear acquisition and extinction.	[123]
				Ext. acquisition	<		
	Healthy right-handed subjects	Amygdala	Right	Ext. acquisition	<	Establish new associations	[126]
			Left		>	Encode fear memories	
		Hippocampus	Left	Ext. acquisition	>		
				Fear acquisition	<	–	
	Healthy subjects	vmPFC	Bilateral	Fear acquisition	>	–	[133]
				Late ext.	<	Mediate extinction recall	
				Ext. recall	<		
		Hippocampus		Ext. recall	<		
		Amygdala		Fear acquisition	<	–	
				Late ext.	<	–	
	Healthy subjects	Amygdala	Left	Fear acquisition	<	Involved in the function of the auditory cortex	[138]
				Early ext.	<		
				Late ext.	<		
		vmPFC	Bilateral	Fear acquisition	>	Recall auditory fear information	
				Ext. acquisition	<		
		dACC		Fear acquisition	<	Maintain fear expression	
				Ext. acquisition	<	–	
		Auditory thalamus		Fear acquisition	<	Maintain durable associations	
				Ext. acquisition	>		
		Primary auditory cortex		Fear acquisition	<	–	
				Ext. acquisition	<	–	
	Healthy female subjects	Anterior insular	Bilateral	Fear acquisition	<	Predict intrusive memories	[135]
				Ext. acquisition	<		
		dACC		Fear acquisition	<		
				Ext. acquisition	<		
				Ext. recall	>		
	PTSD patients	Anterior hippocampus-amygdala regions	Bilateral	Fear acquisition	<	–	[129]
		mPFC				–	
		Anterior hippocampus-amygdala regions		Ext. acquisition	<	Exaggerate threat response	
				Ext. recall	<		
		Medial prefrontal areas		Ext. recall	<	Regulation deficiency	
		Thalamus		Fear acquisition	>	Regulate arousal and awareness.	
				Ext. acquisition			
				Ext. recall			
	PTSD patients	dACC	Bilateral	Fear acquisition	<	Response to cue-elicited fear	[136]
				Early ext. acquisition	<		
				Early ext. recall	<		
		vmPFC		Late ext. acquisition	<		
				Early ext. recall	<		
Two-day Virtual Reality Fear Extinction Experiment	Trauma-exposed children	dACC	Bilateral	Ext. recall	<	Developmental disabilities in extinction neural circuity	[246]
		Anterior insula	Left		<		
Fear conditioning-extinction with 3D virtual reality stimuli	Healthy right-handed subjects	Cerebellum lobule VI	Right	Fear acquisition	<	Proximal threats extinction and predict fear reinstatement	[132]
				Ext. acquisition			
				Fear reinstatement			
		Amygdala-mPFC		Late ext.	<	–	
Fear conditioning-reconsolidation- extinction paradigm	Healthy right-handed subjects	Amygdala	Bilateral	Late ext.	<<	Extinction learning	[131]
		vmPFC-amygdala		Early ext.	>	–	
				Late ext.	<	–	
Three-day retrieval-extinction protocol	Healthy subjects	IT	Right	Ext. recall	>	–	[137]
		dlPFC	Right			–	
		dlPFC-ACC	Bilateral			–	
		IT-dlPFC				–	
		vmPFC		Early ext.	>	–	
				Late ext.	>	–	
Fear conditioning with cognitive emotion regulation instructions	Healthy right-handed subjects	dlPFC	Left	Ext. acquisition	<	Regulate fear	[127]
		Middle frontal gyrus				–	
		vmPFC				–	
		Posterior insular cortex			>	–	
		Amygdala				–	
		Cingulate regions				–	
		dmPFC				–	
Cognitive associative learning paradigm	Healthy subjects	Cerebellar cortex lobule VI	Right	Fear acquisition	<	Fear expression	[247]
				Ext. acquisition	<		
Novelty-facilitated extinction paradigm	Healthy subjects	vmPFC	Bilateral	Ext. acquisition	<	Enhance ext. learning	[124]
				Ext. retention	<	–	
		Thalamus		Ext. acquisition	>	–	
				Ext. retention		–	
		Insula	Left	Ext. acquisition	>	–	
				Ext. retention		–	
		dACC	Bilateral	Ext. acquisition	<	–	
		Superior frontal gyrus	Left	Ext. acquisition	<	–	

<, increased activity; >, decreased activity; Ext, Extinction; vmPFC, ventromedial prefrontal cortex; dmPFC, dorsomedial prefrontal cortex; dlPFC, dorsolateral prefrontal cortex; dACC, dorsal anterior cingulate; IT, inferior temporal cortex

### Understanding Their Relationship and Mechanisms

#### Historical Opinions on Their Relationship

Based on the above evidence, there appear to be competing mechanisms between reconsolidation and extinction [140]. These discrepancies suggest that methodological variables influence fear memory retrieval outcomes. Earlier, Nader et al. found that intra-amygdala infusion of anisomycin (ANI) has an amnesic effect on consolidated memory during the reactivation period in the fear conditioning paradigm [18]. Nevertheless, some findings generated contrasting opinions regarding the effect of ANI on memory retrieval. One group used a one-trial IA task to examine the role of protein synthesis in the hippocampus during extinction memory. As extinction training progresses, the CR of rats gradually diminishes, while a bilateral infusion of ANI into the CA1 region before the extinction retention test completely blocks extinction memory [141]. Furthermore, though infusing ANI into the hippocampus before the first memory retention test impairs memory retrieval at 24 h, it is resistant in the following test sessions, suggesting that the protein inhibitor impairs extinction without affecting consolidated fear memory. The literature contains several inconsistencies, which arise from differences in experimental paradigms, targeted brain areas, and investigators. It is arguable that other studies using similar paradigms in either the amygdala or hippocampus reach the same conclusion as the study of Nader et al. [93], while others suggest that amnesic agents only affect extinction [142].

To reconcile the conflicting findings, several studies have established that reconsolidation and extinction are dissociable on the dominant memory trace (Fig. 3C). The *Chasmagnathus* model of contextual memory has been used to examine CSM with various contextual exposures, ranging from 5 to 120 min [58]. CSM retention tests showed detectable memory for exposure durations < 60 min, regardless of the contextual environment. For exposure durations > 60 min, CSM is significant in the novel context, but not in the training context, suggesting well-established extinction memory. In addition, they used the protein synthesis inhibitor cycloheximide (CHX) and revealed that after exposure, CHX impaired either reconsolidation or extinction in the 5- or 60-min context exposure groups. These results suggest that reminder duration acts as a switch in determining the outcome of memory retrieval and that reconsolidation and extinction are not mutually exclusive but depend on trace dominance.

Later discoveries refined these findings by showing that non-reinforcement and CS offset are necessary for reconsolidation and extinction [103]. One-trial VDS during re-exposure, with CHX given either 2 h later or immediately after re-exposure, failed to affect reconsolidation. However, re-exposure without one-trial VDS did affect reconsolidation, implying that a US before the CS strengthens reconsolidation, preventing amnesic effects even within the effective time window of CHX. Similar experiments on extinction memory confirmed that non-reinforcement significantly impacts memory retrieval outcomes. These findings contribute to an ineluctable interpretation that it is the non-occurrence of the expected reinforcement, terminated by non-reinforcement, that switches reconsolidation to extinction. Pérez-Cuesta et al.’s research modified the CSM retention paradigm by re-exposing rodents in a training context for 15 mins plus an additional 2 h. They tested reconsolidation before the end of the long exposure and assessed extinction memory afterward. Both reconsolidation and extinction were detectable, indicating that CS-offset initiates extinction even when reconsolidation can be tested during long exposures [143].

Nader et al. pointed out that extinction cannot prevent reconsolidation [144], as inhibiting protein synthesis in the BLA of rodents impairs both recently reactivated and extinguished fear memories. Reconsolidation and extinction memory develop in parallel and can overlap, depending on the number of CS presentations [145]. Pérez-Cuesta et al.’s further experiments [143] showed that pre-exposure administration of CHX and varying re-exposure conditions to CS or CS-US confirmed that reconsolidation initiates earlier than extinction memory, with partial overlap (Fig. 3C). This aligns with Nader et al.’s findings [144], emphasizing that reconsolidation cannot be attenuated to facilitate extinction [146]. There are amnesic effects on context-shift procedures if ANI is given after reactivation. However, the results demonstrated no fear of memory renewal in either the same or a different context, indicating that post-reactivation administration of ANI takes effect on the memory storage trace rather than extinction memory, which does not support this hypothesis that reconsolidation can be attenuated to facilitate extinction.

From a molecular perspective, evidence is mixed regarding the distinctiveness of reconsolidation and extinction pathways. Some studies suggest that these processes have separable biochemical signaling pathways. For instance, pharmacologically inhibiting CB1-R and LVGCCs affects extinction but not reconsolidation in CFC. Conversely, factors such as protein synthesis and N-methyl-D-aspartate receptors (NMDARs), which impact reconsolidation, also impair extinction [147]. Supporting this, the expression of the immediate-early genes *Zif268* and *Arc* is dependent on reconsolidation. Conditional knockdown of these genes combined with protein inhibition results in a blockade of reconsolidation while altering their expression prevents extinction. The above evidence demonstrates that reconsolidation and extinction have distinct molecular signatures.

Some reports have demonstrated that reconsolidation and extinction share predominantly identical molecular characteristics (Fig. 3C). For instance, disruption of CREB-mediated transcription blocks both reconsolidation and extinction in CFC [93]. Immunohistochemical analysis of the transcription factor CREB and the expression of *Arc* have revealed significant *Arc* expression in the amygdala and hippocampus during reconsolidation, and in the amygdala and PFC during extinction. Therefore, both processes engage new gene expression in various brain regions, indicating potential interaction between reconsolidation and extinction under certain conditions. Another biomarker in the BLA is calcineurin, which increases during extinction [148]. Its expression pattern remains unchanged even with an NMDA-type glutamate receptor agonist or antagonist, suggesting an insensitive state, termed the “limbo state” where neither process is engaged.

To clarify the concept of the “limbo state” between reconsolidation and extinction, researchers investigated the ERK1/2 signaling pathway in the CFC paradigm. Memory retrieval sessions were divided into four groups based on the number of non-reinforcement sessions (1, 4, 7, and 10) [149]. Minimal CS stimuli (1) represented reconsolidation, and maximal stimuli (10) represented extinction, with intermediate states at 4 and 7 CS stimuli. Intra-BLA administration of the ERK1/2 pathway inhibitor U0126 did not affect memory retrieval during 4/7 CS stimuli, but it did during 1/10 CS stimuli. In addition, manipulating the ERK1/2 pathways with the NMDAR partial agonist D-cycloserine supports the existence of a “limbo state” [62]. This state is a linear progression between reconsolidation and extinction, differing from Pérez-Cuesta et al.’s idea of overlapping reconsolidation and extinction (Fig. 3C). Similar results were found in the IA paradigm, where the ERK signaling pathway was specifically activated during extinction, and CREB expression was anticipated in both reconsolidation and extinction [71]. Since the ERK pathway is upstream of CREB, its activation facilitates extinction while preventing reconsolidated memory, further establishing the “limbo state”. Moreover, a previous study identified “fear neurons” during non-reinforcement reactivation and “extinction neurons” during extinguished memory in the amygdala [150], raising a question about the diversity of neuronal populations involved in fear memory retrieval. Further identification of different neural types responsible for distinct memory stages is necessary.

In summary, reconsolidation and extinction share similar biochemical signatures, but the exact mechanism of how reconsolidation turns into extinction remains largely unknown.

#### Engram Cells in Memory Reconsolidation and Extinction

Reconsolidation and extinction are both memory-updated processes triggered by the presence of the CS [146], involving new protein synthesis. In addition, these two mnemonic processes share partially-overlapping molecular and neuronal profiles. Questions arise: What proteins are synthesized? Where is the updated memory stored? Are there two phenotypically distinct populations that regulate reconsolidation and extinction separately? Richard Semon’s Engram Theory may help explain these discrepancies. Proposed over a century ago [151], it was not until the turn of the millennium that the “engram cells” theory gained significant attention in the study of memory owing to the technical restrictions of that era [152]. The term “engram” refers to an organic substance that stores memories with three features: (1) activation by a learning experience; (2) modification by an artificial learning experience; and (3) reactivation by subsequent retrieval, often involving non-engram, silent, and active engram cells [151, 153].

The relationship between memory consolidation and engrams remained unexplored until researchers found that protein synthesis inhibitors can induce amnesia, which can be reversed by artificially activating the engram cells labelled during fear training. This phenomenon can also occur when amnesic treatments are administered during the reconsolidation period [154]. During CS retrieval, hippocampal engram cells activated by backward conditioning (US-CS) are labeled, showing that CS retrieval can significantly increase the overlapping proportion of the fear conditioning engram and retrieval-induced engram cells, indicating a reactivation of the fear conditioning engram. This indirect retrieval procedure also underlies the reconsolidation phase, since amnesic treatment into the hippocampus during a post-reactivation period can interfere with the original fear memory [155]. Further, optogenetic activation of c-*Fos*-tagged dDG ensembles impairs conditioned fear responses during post-reactivation [66], regardless of the valence of the engram. Nevertheless, this freezing decline phenomenon can be replicated if a sufficient proportion of dDG neurons are activated, indicating that the reconsolidation process is not linked to engrams of a specific valence. Furthermore, the reconsolidation process involves different engram cells for fear conditioning and reinstatement, with minimal overlap, implying that reconsolidation re-engagement of fear conditioning engram ensembles may generate new engrams. The verification can be expanded in the amygdala where *c-Fos-LacZ* transgenic rats in the auditory FC paradigm [156] showed that high neuronal activation leads to the expression of c-*Fos* and LacZ mRNA, followed by β-galactosidase (β-gal) protein expression. Infusion of Daun02, which disrupts neuronal function when converted to Daunorubicin by β-gal, significantly decreased freezing levels after reactivation, demonstrating that reconsolidation reactivates original fear conditioning engrams rather than creating new engram ensembles. Thus, manipulating reconsolidation engram cells may be less effective, as reconsolidation primarily reactivates existing fear conditioning engrams.

Extensive evidence has shown the widespread existence of extinction engram cells throughout the brain [115, 119, 157–161]. Fear extinction engrams, identified by engram cell labelling technology in transgenic rodents, facilitate the labelling and manipulation of activated neurons across different mnemonic periods. Extinction activates engram ensembles in the dDG, which can be artificially disrupted during extinction retrieval [159]. Moreover, the genetical signature of the amygdaloid extinction engram ensembles has been identified in *Ppp1r1b*^+^ BLA neurons [160]. Building upon the “prediction error” theory, it is tempting to speculate that exposing engram cells to a gradually decreasing frequency of non-reinforced stimuli could encode diverse information. Attempts to reverse memory valence can be made by reactivating original engrams while applying conditioning of opposite valence [162], potentially converting related information from negative to positive. Interestingly, in both contextual fear and reward conditioning, engram cells in the dDG encode bidirectional valence with a switch in functional connectivity due to progressively growing synaptic strength [154]. Also, the reward-responsive neurons exhibit functional equivalence with the extinction engrams. This implies that extinction learning represents a positive rewarding experience for fear-conditioned subjects [66]. The engram cell theory provides a cornerstone for explaining memory malleability.

**Fig. 4:**
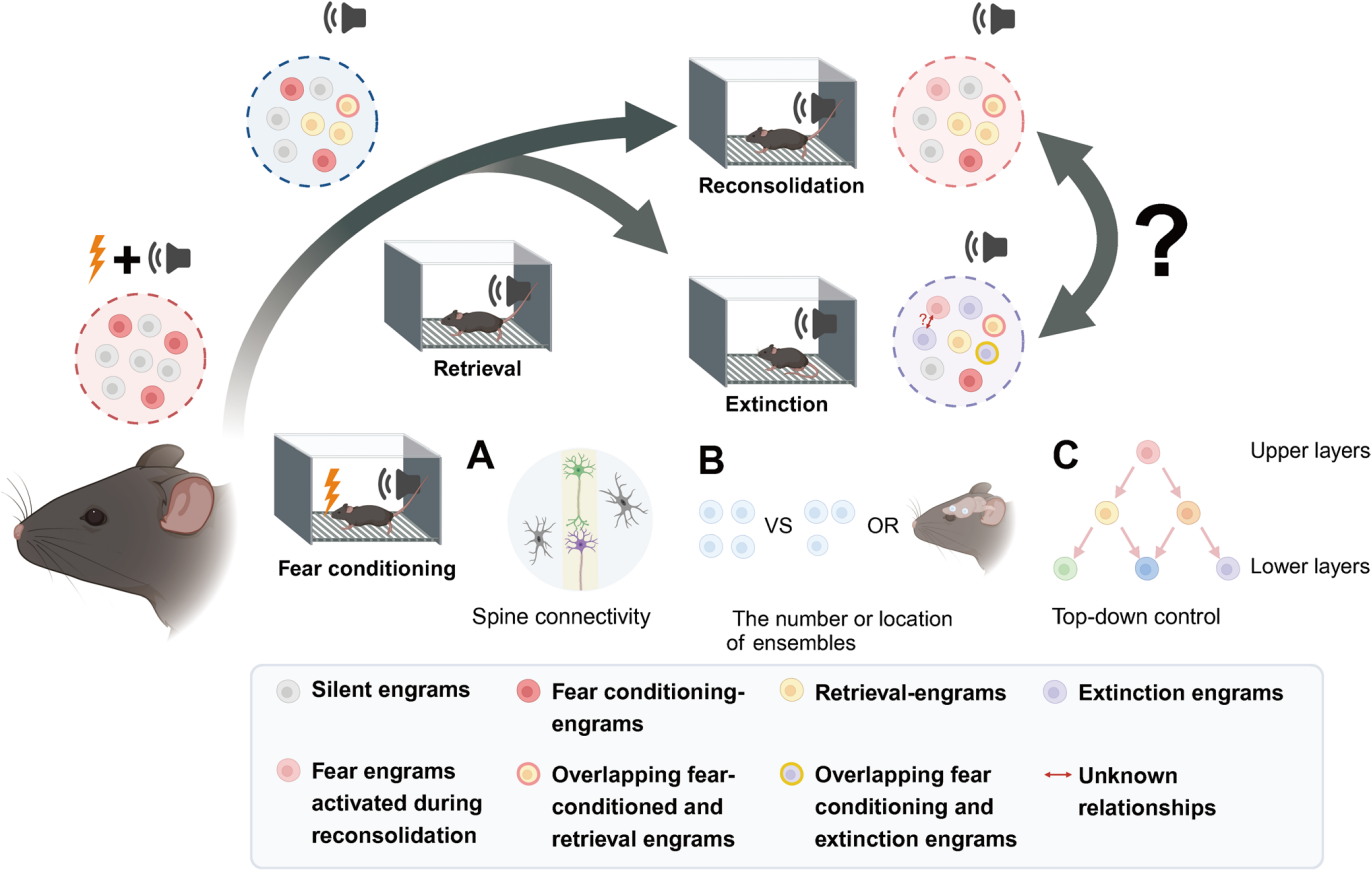
A possible role for engram cells pertaining to memory reconsolidation and extinction. This review proposes the existence of distinct engram ensembles during mnemonic stages, such as reconsolidation and extinction. During fear conditioning, a cohort of silent engram cells (grey, silent engrams) encode new information and become conditioning-activated engram cells (scarlet, fear conditioning engrams). Upon memory retrieval, other engram ensembles are activated by the CS (yellow, retrieval engrams). Some conditioned- and retrieval-engrams may overlap (yellow with red border, overlapping fear-conditioned and retrieval engrams). Memory retrieval triggers two opposing stages, namely reconsolidation and extinction. The former process reactivates original fear conditioning engrams (red, fear engrams activated during reconsolidation), and the latter process generates new engram ensembles (violet, extinction engrams). As extinction learning progresses, some fear engrams may switch into extinction ensembles (violet with yellow border, overlapping fear conditioning, and extinction engrams). However, the intrinsic switch mechanism between reconsolidation- and extinction-engrams is not well understood (black question mark). Potential factors govern the competition between reconsolidation and extinction engram cells including spine connectivity **(A**), the number or location of engram ensembles **(B**), or projections from upper brain structures **(C**). The hypothetical mutual interaction between reconsolidation- and extinction-engram cells requires further exploration (tiny red question mark, unknown relationships)

Several studies have revealed that fear acquisition engrams can be suppressed by extinction engram cells, highlighting distinct neuronal ensembles governing fear memory [115, 158, 159]. During the memory updating process initiated by non-reinforced stimulus-initiated fear engrams increasingly overlap with extinction engram cells in the PL [161]. These findings suggest that as extinction learning progresses, some fear engrams may switch into extinction ensembles (Fig. 4). Despite evidence that activated engram cells often inhibit the recruitment of non-activated ones [163, 164], whether a competitive mechanism among engram cells is triggered by the PE remains elusive. Following this question, scientists have demonstrated that it is the “collocation window” between events that establishes competition among engram cells in the lateral amygdala, regulated by parvalbumin interneurons that release GABA [165]. When rodents receive two distinct fear conditioning events within 6 h, engram cells integrate collaborative memories. However, for events >1 day apart, engram cells represent them as independent events. This discovery is consistent with the concept of the “reconsolidation window” [33], which delineates the effective timeframe for rewriting memory. In this context, if the non-reinforcing stimulus is presented during the reactivation period, the already-formed associations in the fear engram cells are overwritten in the absence of the US. If non-reinforcing stimuli are presented during reactivation, the existing associations in fear engram cells may be overwritten in the absence of the US [161]. Fear-related learning mechanisms form strong connections among fear engrams, but concerted efforts are required to degrade these neural associations. Engrams encoding different memories can be reflected in their morphological changes through spine connectivity or vector distance within the population [154, 161]. Caution is warranted regarding the potential of silent engram cells. Emerging evidence supports the hypothesis that it is the silent-state, rather than the latent state, of engram cells in a silent-state, not a latent-state, that makes them inaccessible under natural conditions. However, artificial regulation may facilitate access to these cells, thereby reducing the likelihood of repeated CS exposures activating the dormant engrams [157, 159, 166].

## Behavioral Therapy Based on Reconsolidation

Currently, ET aims at weakening traumatic memory associations by inducing reconsolidation and has emerged as a promising alternative to the standard ET strategy. This approach updates memory by decreasing amygdaloid activity [167, 168], thereby reducing the emotional impact of traumatic memories. By presenting unreinforced CSs within this labile, retrieval-induced period, memory is updated so that the CS, once predictive of danger, is now associated with the absence of the US. This shift can potentially lead to the permanent erasure of fear memories without the use of medications [32]. Known as the “retrieval-extinction” the “post-retrieval extinction” (PRE) protocol, or the “reconsolidation of traumatic memories” (RTM) protocol [169–171], this method has advantages over traditional extinction therapy, such as cost-effectiveness and high adherence rates; it is gaining popularity among ET alternative [172, 173]. For limitations of ET, refer to Table 6.

**Table 6:** Limitations of exposure therapy among PTSD patients

Type of exposure therapy	Subjects	Evaluation standard	Course of treatment (weeks/m)	Follow-up sessions	Treatment dropout (%)	Therapeutic outcomes	Limitations	Possible reasons	References
CPT	94 active service duty members with a diagnosis of PTSD and MDD	a. MADRS b. CAPS-5 c. PHQ-9 d. PCL-5	20	3 weeks	15	No comparable significant difference.	The use of BA may not be necessary over CPT alone.	Treatment options may be decided by patient preference and shared decision-making	[248]
CPT+BA					21				
D-CPT	Adolescent PTSD patients (aged 14-21 years, *n*=38)	a. Self-developed TAS b. Self-developed TCS c. HAQ d. CAPS-CA	16-20	3 weeks	13.63	Therapeutic alliance is positively correlated with reduced PTSD symptom	Therapeutic adherence and competence are negatively correlated to the treatment outcome	Lack of therapist adherence and competence	[249]
GBCT	PTSD veterans (*n*=98)	a. CAPS-5 b. BDI-II and BAI c. SCID-IV-TR d. TLEQ e. SF-36 f. CERQ-Short	16	–	38.4	–	Treatment noncompletion	Reported lifetime trauma exposure, higher depression symptoms.	[250]
GPCT	PTSD veterans (*n*=100)				20.2	2.389 times more likely to compete for treatment than GBCT groups	–	A group therapy strategy is preferred	
PE delivered *via* video teleconferencing	Rural veterans (*n*=27)	a. PDS b. BDI c. BAI d. CAPS	12	1 month	70 in the iPhone group	Decrease PTSD symptom	Significant high attrition	Poor technical support	[251]
PE	PTSD male combat veterans (age=65 years, *n*=87)	a. CAPS b. SCID-IV-TR c. PCL-S d. PHQ-9	12	6 weeks	27	PTSD symptomology has improved	Hard to maintain therapeutic outcomes	Remote traumatic memories are resistant to interference	[252]
RT									
MT	PTSD veterans (*n*=370)	a. PSS-I b. PCL-S	2	6 weeks	13.6	No extinguished treatment found	Trauma-focused treatment may not be suggested for improving PTSD symptoms in active-duty military personnel.	Alternative treatments may be needed to investigate better clinical outcomes.	[253]
ST			8		24.8				
PCT			8		12.1				
PE with AE (imaginal exposure)	Active duty United States service members (*n*=125)	a. PCL-S b. PSS-I c. BDI-II d. BAI e. STAXI-2 f. ADUIT g. SSI	8–12	1 and 6 months	52.8	Not as ideal as delivered PET alone	AE intervention fidelity is not systemically assessed post-treatment	Personalized psychotherapy is recommended	[254]
3MDR	Treatment-resistant PTSD veterans (*n*=43)	a. CAPS-5 b. PCL-5 c. PABQ	6	12 and 16 weeks	7	45% of patients improved clinically	No statistical variations of PTSD symptom severity between groups	A larger sample size and wider clinical application are needed	[9]
VRE	Active duty soldiers with PTSD (*n*=108)	a. SUDS b. CAPS	26	3 and 6 months	44	A greater decrease in CAPS scores without group difference	Poor emotional engagement	Substantial emotional and stress evaluation should be established	[255]
PE					41		-		
VRET	Active duty soldiers with PTSD (*n*=153)	a. CAPS	9	3 months	16.2	CAPS-score improved 31%	High dropout occurred only in the VRET group	A common phenomenon that is consistent with other reports	[256]
CRET					0	CAPS-score improved 37%	–	–	
TMT	Veterans and active duty personnel with PTSD (*n*=112)	a. CAPS b. PCL-M c. SCID I and II d. SF-36 e. BRIEF-A f. TRGI g. CGI h. HAMD	17	3 and 6 months	2	High-level and satisfactory treatments	Not a randomized controlled trial	Latent variables should be constructed	[257]
Web-PE	40 military personnel with PTSD	a. CEQ b. CAPS-5 c. PCL-5 d. PHQ-9 e. VR-12	8	3 and 6 months	52.6	Reduced PTSD symptom	High dropout rate	Achieve better therapeutic outcomes in open trial participants.	[258]
PCT					23.8	No comparable difference, but PCT achieved clinically significant change.			
NET	Patients with comorbid BPD and PTSD (*n*=11)	a. SCID-I and II b. CTQ	10	12 months	9.1	NET is feasible and safe in an inpatient program	Lack of outpatient use	This type of inpatient setting is common in Germany	[259]
DET	PTSD patients (*n*=141)	a. IDCL b. IES-R c. PDS d. BSI e. PTCI f. IIP-C g. Global Severity Index	Not mentioned	6 months	12.2	CPT performed significantly better than DET	Unreliable remission rates	Incomplete diagnostic criteria for PTSD symptoms	[260]
CPT					14.9				

3MDR, virtual reality and motion-assisted exposure therapy; AE, aerobic exercise; AUDIT, Alcohol Use Disorders Identification Test; BA, behavioral activation; BAI, Beck Anxiety Inventory total score; BDI-II and BAI, the Beck Depression Inventory-II and Beck Anxiety Inventory; BPD, Borderline Personality Disorder; BRIEF-A, the Behavior Rating Inventory of Executive Function-Adult Version; BSI, Brief Symptom Inventory; CAPS-5, the Clinician-Administered PTSD Scale for DSM-5; CAPS-CA, the German version of the Clinician-administered PTSD Scale for Children and Adolescents; CEQ, Credibility/Expectancy Questionnaire; CERQ-Short, the Cognitive Emotion Regulation Questionnaire-Short Form CPT, cognitive processing therapy; CGI, Clinical Global Impressions Scale; CHANGE, a coding system designed to capture processes of therapeutic change, including both facilitators and inhibitors of change; CPT, cognitive processing therapy; CRET, control exposure therapy; CTQ, Childhood Trauma Questionnaire; D-CPT, developmentally adapted cognitive processing therapy; DET, dialogical exposure therapy; GBCT, group cognitive behavioral treatment; GPCT, group present-centered treatment; HAQ, the German version of the Helping Alliance Questionnaire; HADS, Hospital Anxiety and Depression Scale; HAMD, Hamilton Rating Scale for Depression; IDCL, the German version of the International Diagnostic Checklist for DSM-IV and ICD; IES-R, Impact of Event Skala-revidierte Version; IIP-C, the Inventory of Interpersonal Problems-Circumplex Version; MADRS, Montgomery-Åsberg Depression Rating Scale; MDD, major depression disorder; MT, massed therapy includes prolonged exposure therapy, cognitive behavioral therapy involving exposure to trauma memories/ reminders are administered altogether; NET, Narrative exposure therapy; PABQ, Posttraumatic Avoidance Behaviour Questionnaire; PCL-5, PTSD Checklist for DSM-5; PCL-M, PTSD Checklist-Military Version; PCL-S, PTSD Checklist-Stressor-Specific Version; PCT, present-centered therapy; PDS, a self-report instrument which can be used for determining PTSD diagnostic status according to DSM-IV criteria and symptom severity; PE/PET, prolonged exposure therapy; PHQ-9, Patient Health Questionnaire-9; PSS-I, PTSD Symptom Scale-Interview; PTCI, Posttraumatic Cognitions Inventory; SCID I and II, the Structured Clinical Interview for DSM-IV to assess other Axis I and II disorders; SCID-IV-TR, the Structured Clinical Interview for DSM-IV-TR Axis I Disorders-Patient Edition with Psychotic Screen; SF, the Physical Role Functioning subscale of the Medical Outcomes Study Short-Form Health Survey; SF-36, Health-Related Functioning: Medical Outcome Study Short Form-36 Health Survey; SSI, Scale for Suicidal Ideation; ST, spaced prolonged exposure therapy; STAXI-2, State-Trait Anger Expression Inventory; SUDS, subject units of discomfort; TAS, therapeutic adherence scale; TCS: therapeutic competence scale; TRGI, Trauma-Related Guilt Inventory; TLEQ, the Traumatic Life Events Questionnaire; TMT, trauma management therapy; VR-12, the Veterans RAND 12-Item Health Survey; VRE/VRET, visual reality exposure therapy; Web-PE, a web version of Prolonged Exposure Therapy

Several factors contribute to the efficacy of reconsolidation-based protocols. Firstly, it is the timing that matters. The success of this intervention relies heavily on the time window during which memories are temporarily destabilized and susceptible to modification. In humans, reactivating fear memories 10 min, but not 6 h, before extinction produces a durable reduction in fear that can last at least a year [33]. Secondly, a degree of PE is essential for destabilizing memories. Multiple PEs weaken original fear memories more effectively and prevent fear from returning during reinstatement compared to a single-PE strategy [174]. This protocol leverages PE to create a discrepancy between the original learning context and the reactivated context [175]. Moreover, an uncertain number of PEs, rather than a PE alone, may more effectively prevent fear return under a laboratory paradigm [176]. The above evidence suggests that the degree of violation during the memory-updating process facilitates fear attenuation and extinction learning. Thirdly, the unpredictability of interval length is also vital. In a PRE training paradigm, researchers found that distributing random inter-trial intervals (ITIs) during the reactivation, rather than fixed ITIs, leads to a greater erasure of fear memory [177]. Greater variability in ITIs results in a more significant erasure of reconsolidated memory [178]. Finally, PTSD patients harbor persistent fear memories that limit the effectiveness of standard ET. Thus, preventing the recurrence of old fear memories is an important measure of PRE therapy’s robustness. Human studies have shown that PRE can effectively eliminate both recent (1-day-old) and remote (7- and 14-day-old) fear memories [179, 180]. A modified PRE protocol, in which the US is presented before extinction, prevents fear relapse for at least 6 months [179]. Encouragingly, this approach has also shown success in treating naturally-occurring phobias, like arachnophobia [167, 181], and offers promise for those with severe, prolonged trauma exposure [171, 172, 182]. As females are more susceptible than males [183], reconsolidation-based protocols have shown promising results in female patients as well [182]. In sum, these studies provide compelling evidence that reconsolidation-based protocols are not only effective in PTSD, but also offer long-lasting prevention against the return of fear.

Some failures in testing the efficacy of the reconsolidation-based treatments in laboratory-induced fear cannot be overlooked [34, 169, 170, 184], potentially due to the instability of engram cells. Engrams stabilize during consolidation. Stable engrams can be consistently reactivated during both recent and remote memory retrieval, and this persistence is vital for memory maintenance [185]. This rigidity is maintained through mechanisms such as DNA methylation [186] or synaptic strength and spine density augmentation [154]. First, the intensity of extinction training may be insufficient. Experimental fear paradigms applied in healthy participants often span 3 to 4 days, including the extinction training phase, and differ significantly from real-world fear scenarios. Consequently, the artificially-induced extinction memory may fail to establish stable engram cells, leaving it as a fragile, temporary trace unable to counteract the engram connections of the original fear memory because increased ensemble stability is vital for memory permanency [185]. Second, changes in experimental conditions, such as replacing the original aversive stimuli with neutral ones, can contribute to failure. Third, individual differences may cause variability in extinction learning, preventing the elimination of fear memories. Furthermore, the use of medications in some paradigms introduces further uncertainty compared to purely behavioral approaches.

## Perspective

PTSD is a mental health disorder inflicted by traumatic experiences, leading to flashbacks, avoidance, and cognition impairments that disrupt daily life [187]. Understanding the pathogenesis of PTSD at the biomolecular and neuronal circuit levels may offer new therapeutic insights. While the role of engram cells in the switch between reconsolidation and extinction remains uncertain, increasing research underscores their importance in both processes, providing a springboard for future work. A key question is what factors govern the competition between reconsolidation and extinction engram cells. Potential influences include spine connectivity, ensemble number, location, and projections from upper brain structures (Fig. 4A-C), though current evidence does not fully explain these assumptions. Achieving these advances requires a detailed mapping of neurocircuitries, supported by technologies such as fMRI and comprehensive electroencephalogram monitoring. In preclinical research, distinct molecular targets involved in memory destabilization are identified by activating encoded engram cells under specific conditions. Techniques such as single-cell sequencing and neural activity-dependent labeling in animal models of PTSD could help uncover biomarkers associated with reconsolidation and extinction engram cells.

For human studies, a standardized protocol should first be developed. Boundary conditions, such as the type of fear memory, memory age, and intensity of stimuli, should closely replicate original memory scenarios to effectively engage engram cells. Engram stability is closely tied to memory context, emphasizing the need to replicate the encoding scenario. Script-driven imagery and music elicit emotional responses to disrupt memory reconsolidation. Interactive tools, like computer games or Virtual Reality (VR) technology, show significant promise as an intervention for PTSD [188]. By generating vivid and immersive artificial environments, VR can effectively incorporate cues associated with individual traumatic experiences, thereby facilitating a more personalized and potentially therapeutic approach to treatment. Multi-center, larger-sample studies are essential to validate the robustness of this methodology. In addition, computer science can predict the outcomes of reconsolidation-based interventions by developing metacognitive frameworks to predict how memory-updating factors may influence the outcomes [189, 190]. Artificial models help further overcome the obstacle of small clinical populations by enabling a better understanding of how individual patient profiles, including etiology, brain circuity, genetic variations, and sex differences modulate the interplay of boundary conditions, identify general patterns, and optimize future treatment strategies.

A key milestone is identifying engram cells in the human brain. Using fMRI data during reactivation may identify brain regions harboring engram cells. It is imperative to develop a behavioral marker of reconsolidation using fMRI at single-cell resolution [191, 192] that can be used in large-scale clinical applications. From brain regions to cellular biomarkers by combining advanced techniques, major efforts should prioritize bridging the gap from the bench to the bedside. Such efforts will not only broaden our understanding of engram cells in PTSD but also lay the groundwork for developing precise and personalized treatments for trauma-related disorders.
